# Test Experience, Direct Instruction, and Their Combination Promote Accurate Beliefs about the Testing Effect

**DOI:** 10.3390/jintelligence11070147

**Published:** 2023-07-21

**Authors:** Michelle L. Rivers

**Affiliations:** 1 Department of Psychology, Texas Christian University, Fort Worth, TX 76109, USA; m.l.rivers@tcu.edu; 2 Department of Psychological Sciences, Kent State University, Kent, OH 44240, USA

**Keywords:** metacognition, learning strategies, self-regulated learning, testing effect, retrieval practice

## Abstract

Practice testing is a highly robust learning strategy that promotes long-term retention, especially in comparison to more passive strategies such as restudying—a finding referred to as the testing effect. However, learners do not always appreciate the memorial benefits of practice testing over restudying, which could limit their use of practice testing during self-regulated learning. The current investigation explored the extent to which learners’ metacognitive judgments about the testing effect can be improved via test experience, direct instruction, or a combination of both techniques. Prolific participants underwent two learning cycles. In the first cycle, participants were randomly assigned to either (a) experience a testing effect in their own memory performance (i.e., study unrelated word pairs, practice half the pairs through restudying and half through testing with correct-answer feedback, complete a critical test on the pairs, and receive feedback regarding their performance after using each strategy); (b) imagine they had to learn word pairs and read a passage on the purported benefits of practice testing; or (c) undergo both procedures. In the second cycle, all participants learned a novel set of word pairs. Across both learning cycles, participants estimated memory performance for material learned through testing versus restudying. Both test experience and direct instruction—independently and in combination—led to more accurate memory estimates across learning cycles, but no technique was more effective than the other. In summary, people can learn about the memorial benefits of practice testing when they experience a testing effect on their own memory performance and/or when they receive instruction about its benefits.

## 1. Introduction

To be successful in educational contexts, students must be effective self-regulated learners, i.e., students must be motivated, reflective, and proactive in their use of effective learning strategies and be able to apply them appropriately (Bjork et al. 2013; Panadero 2017). One such strategy that consistently leads to lasting learning is practice testing (Dunlosky et al. 2013). For example, consider an investigation conducted by Roediger and Karpicke (2006). College students read a short passage on “Sea Otters” or “The Sun”. Following the initial reading, students were then randomly assigned to either reread the passage or take a practice test on the passage (i.e., recall as much of the passage as they could from memory and write it down). Following a delay of either 5 min, 2 days, or 1 week, students received a final test on the passage in which they were asked to write down as much as they could remember from the passage. For students who received the final test after 5 min, memory performance was higher for those who reread the passage compared to those who received a practice test on the passage. In contrast, for students who received the final test after a longer delay of 2 days or 1 week, performance was higher for those who received a practice test compared to those who reread the passage.

This latter finding—in which long-term memory performance is higher for material that is practice tested compared to material that is reread or restudied—is referred to as the testing effect (sometimes called the retrieval practice effect or test-enhanced learning). The testing effect has been demonstrated across a wide variety of materials, learners, contexts, and outcome measures (for recent reviews, see Agarwal et al. 2021; Carpenter et al. 2022; Moreira et al. 2019; Yang et al. 2021). However, in a comprehensive review of over 100 papers investigating learners’ beliefs about and use of practice testing, Rivers (2021) concluded that students do not always appreciate the benefits of practice testing for lasting learning and do not use it as effectively as they could. For example, multiple investigations have assessed learners’ beliefs about the testing effect by soliciting metacognitive judgments during learning. In these experiments, learners study material, practice some of the material through testing and some through restudying, then make global-differentiated predictions about how much material practiced with each strategy they will remember on a final test (usually administered after a delay). Although most learners demonstrate a testing effect in their memory performance on the final test, their predictions indicate that they believe restudying is equally effective or even more effective than practice testing (e.g., Agarwal et al. 2008; Einstein et al. 2012; Kang 2010; Kirk-Johnson et al. 2019; Kornell and Son 2009; Roediger and Karpicke 2006; Tse et al. 2010; Tullis et al. 2013)—a metacognitive illusion. Thus, inaccurate beliefs about relative strategy effectiveness is one barrier to students’ adoption of practice testing during their own self-regulated learning, which could limit their academic achievement. Accordingly, the current investigation aimed to improve learners’ beliefs about the relative effectiveness of practice testing versus restudying for long-term memory to inform educational interventions focused on promoting student success.

Accurate information about strategy effectiveness can come from multiple sources, such as direct instruction from an educator (McCabe 2011), or through experiencing the benefits of the strategy on one’s own memory performance (Tullis et al. 2013). In the current research, I compared these two techniques. Specifically, I aimed to answer the following question: To what extent can learners’ beliefs about the testing effect be improved via test experience, direct instruction, or a combination of both techniques? To examine learners’ general beliefs about practice testing and restudying, learners in the current investigation made global-differentiated predictions about memory performance for material learned with each strategy (as in Rivers et al. 2022; Tullis et al. 2013). These judgments were collected across two learning cycles, and changes in predictions before and after test experience and/or direct instruction were assessed. If learners successfully update their beliefs about the relative effectiveness of practice testing versus restudying, they should make higher predictions for tested than for restudied materials during the second learning cycle.

No investigation to date has compared these two techniques for addressing learners’ beliefs about testing as a learning strategy, but there are important reasons why investigating the effectiveness of these techniques—both independently and in combination—is important. Instruction and experience may play different roles in addressing learners’ beliefs about strategy effectiveness. To discern the potential contributions of these two approaches to correcting learners’ beliefs, it helps to understand why learners may hold inaccurate beliefs about the effectiveness of practice testing for learning.

### 1.1. Origins of Learners’ Strategy Beliefs

One reason students may hold inaccurate beliefs about practice testing is that they never receive formal instruction about effective learning strategies. An informative finding in this regard was reported by Kornell and Bjork (2007), who surveyed 472 undergraduates and only 20% of them reported that they study the way they do because a teacher taught them to study that way (see also Hartwig and Dunlosky 2012). In addition, even when students do receive instruction about how to learn, there is no guarantee they are receiving instruction about empirically supported strategies. Teacher-education materials rarely explain the science behind learning strategies such as practice testing (Dunlosky et al. 2013; Pomerance et al. 2016; Surma et al. 2018), suggesting that many educators may never be exposed to them and thus cannot pass this information on to their students. In addition, students’ experience with testing in educational settings—primarily as a means of summative assessment used to assign grades (Morehead et al. 2016; Wasylkiw et al. 2008)—may lead them to view tests as a tool to monitor their learning (e.g., identify gaps in their own knowledge) rather than as a tool to enhance memory (e.g., Gagnon and Cormier 2019; Kornell and Bjork 2007; Tullis and Maddox 2020). To overcome these shortcomings, students may benefit from direct instruction about practice testing as an effective learning strategy.

Learners’ beliefs about testing and restudying may also be reinforced by their objective and subjective experiences during self-regulated learning. Although testing enhances long-term memory compared to restudying, restudying often produces better outcomes on tests of immediate or short-term performance (e.g., Roediger and Karpicke 2006). Thus, if learners use immediate performance as the basis for their beliefs (and thus their metacognitive judgments) about particular strategies, then judging restudying as more effective than testing would be expected (Finn and Tauber 2015). Relatedly, whereas testing can involve failure (i.e., when retrieval is unsuccessful) and feel more effortful (e.g., Kirk-Johnson et al. 2019), restudying may lead to a false sense of fluency (i.e., subjective ease of processing) during learning because the material is readily available, i.e., learning may feel easier with restudying than with practice testing, and prior research suggests that learners use the heuristic “easily learned means easily remembered” when making judgments about future memory (Koriat 2008). Thus, having students experience the effects of practice testing (versus restudying) on their long-term memory may help them recognize that using immediate performance as a basis for their beliefs about strategy effectiveness can lead them to (partially) incorrect conclusions. 

Koriat and Bjork (2006) distinguish between “theory-based” and “experience-based” debiasing techniques, and they argue that both approaches are needed for certain metacognitive illusions. Theory-based debiasing can involve explaining the benefits of a particular strategy (henceforth direct instruction), whereas experience-based debiasing can involve experiencing memorial differences between learning conditions (henceforth test experience). The authors compared these two techniques for addressing the foresight bias—the finding that people predict forward-associated word pairs (e.g., umbrella-rain) as similarly recallable to backward-associated pairs (e.g., rain-umbrella), even though the latter are more difficult to remember on cued-recall tests (e.g., rain–?). They found that only a combination of both debiasing techniques mended this illusion when making memory predictions for a new set of word pairs. Accordingly, because a combination of a priori beliefs and experiences contribute to the metacognitive illusion that restudying is as effective or superior to practice testing for memory, a combination of both instruction and experience may be better than either alone for mending this illusion (for a similar argument, see Yan et al. 2016). To inform the current experiment, I discuss prior research on direct instruction and test experience for the remainder of the Introduction.

### 1.2. When Is Direct Instruction Most Effective?

To address students’ lack of or incomplete knowledge about learning strategies, research has investigated the effectiveness of strategy instruction on measures of students’ beliefs, strategy use, and measures of achievement (for reviews, see Borkowski et al. 1987; De Boer et al. 2018; Donker et al. 2014; Hattie et al. 1996; Pressley et al. 1989). This research provides insight into when strategy instruction is the most effective: Instructions that include elaborated information about why a particular strategy is effective tend to be more effective at changing behavior than simple instructions that exclude such information (e.g., O’Sullivan and Pressley 1984).

For example, Yan et al. (2016) compared three types of instruction for changing people’s beliefs about interleaved practice—in which concepts are mixed during study—versus blocked practice—in which related concepts are presented consecutively. Learners tend to believe that classification performance (e.g., matching a painting with the corresponding artist) is better following blocked practice versus interleaved practice, when the opposite tends to be true. In an attempt to mend this metacognitive illusion, all learners gained experience with both presentation schedules for a set of paintings and then were randomly assigned to read either: (1) no information, (2) information about the percent of people who tend to perform better following interleaved practice (90%), (3) percentage information as in (2) and a statement that acknowledged that blocking often “feels easier”, or (4) information from (2) and (3), plus an explanation for why interleaving is more effective than blocking. After reading this information, participants were asked to select which schedule (blocked or interleaved practice) leads to better classification performance. The more information they were provided during instruction, the more likely they were to hold accurate beliefs—that is, correctly endorse interleaving over blocking (although many learners still maintained the inaccurate belief that blocking was the more effective learning schedule).

In addition to lacking sufficient knowledge about effective strategies such as practice testing, learners may hold misconceptions about particular strategies. As mentioned earlier, many students view testing primarily as a tool to monitor their ongoing learning. Though testing is effective for metacognitive monitoring (e.g., Little and McDaniel 2015), the goal of the current research was to have learners appreciate testing as a tool for enhancing memory. Thus, directly addressing the misconception that testing is solely a monitoring tool may be necessary to change learners’ beliefs. A vast literature on correcting misconceptions has identified effective correction techniques (Ecker et al. 2014; Guzzetti 2000; Guzzetti et al. 1993; Lewandowsky et al. 2012; Tippett 2010). One such technique involves reading a refutation text, which directly states the misconception, negates it, and provides a supporting explanation. Consistent with research on strategy instruction (e.g., Borkowski et al. 1987), refutation texts are most effective at changing beliefs when they include both corrective feedback and an explanation compared to corrective feedback alone (Ecker et al. 2014; Rapp and Kendeou 2007, 2009).

Taken together, research on strategy instruction and correcting misconceptions suggests that effective instruction aimed at improving learners’ beliefs about practice testing should: (a) acknowledge that restudying feels easier than practice testing (i.e., fluency discounting; Yan et al. 2016), (b) address learners’ misconception that testing is solely a monitoring tool, and (c) include an explanation for why testing is more effective (as compared to restudying) for enhancing memory.

Some research has specifically investigated the effectiveness of instruction about practice testing both in laboratory settings (Ariel and Karpicke 2018; Dunlosky and Rawson 2015; Hui et al. 2021a, 2021b, 2022; Maddox and Balota 2012; Simone et al. 2023) and educational settings (Bernacki et al. 2020; Broeren et al. 2021; Einstein et al. 2012; Hotta et al. 2014; Lineweaver et al. 2019, 2022; Luo 2020; McCabe 2011; McCabe et al. 2021; Ploran et al. 2023; Rodriguez et al. 2018), with some finding positive effects on learners’ beliefs and learning behavior. For example, McCabe (2011) found that endorsement of practice testing as a learning strategy was higher for undergraduate students who received targeted instruction about the strategy in their Psychology course compared to those who did not receive instruction, especially when such instruction included a discussion of the empirical evidence supporting strategy effectiveness. In addition, Ariel and Karpicke (2018) found that learners were more likely to engage in practice testing (versus restudying) after receiving instruction emphasizing the mnemonic benefits of the strategy and how to use it compared to a control group that did not receive such instruction (see also Simone et al. 2023). Although these authors did not directly assess learners’ strategy beliefs, learners’ behavior suggests that the instruction was effective at teaching them about the value of using practice testing to enhance memory.

### 1.3. When Is Test Experience Most Effective?

To understand the conditions under which people can learn from experience about the relative effectiveness of particular learning strategies, researchers have used the method of knowledge updating (introduced by Brigham and Pressley 1988). In a typical investigation, learners undergo multiple study–test cycles using different strategies (e.g., learn word pairs using repetition versus imagery) and make metacognitive judgments regarding memory for material studied with each strategy. Although study–test experience alone is not always sufficient to promote accurate beliefs about learning strategies (e.g., Hertzog et al. 2009; Mueller et al. 2015; Pan and Rivers 2023; Price et al. 2008; Tullis et al. 2013), various forms of scaffolding have been found to promote accurate strategy beliefs (e.g., Mueller et al. 2015; Pan and Rivers 2023; Price et al. 2008; Rivers et al. 2022; Tullis et al. 2013; Yan et al. 2016). 

Many of these scaffolds are informed by the theoretical framework proposed by Dunlosky and Hertzog (2000), which outlines specific conditions that must be met for learners to update their beliefs: (a) one strategy must lead to better memory performance than the other strategy (the effectiveness assumption), (b) learners must accurately monitor the differential effectiveness of each strategy during study and/or testing (the monitoring assumption), (c) learners must attribute their differential test performance to the strategies used (the updating assumption), and (d) learners must use their updated knowledge to inform their metacognitive judgments about strategy effectiveness (the utilization assumption). Learners are not always accurate in inferring how many items were remembered on a critical test when using each strategy (i.e., a failure to meet the monitoring assumption), but techniques that make this particular task easier have led to learners updating their strategy beliefs. For example, Price et al. (2008) found that strategy beliefs were more accurate when items learned by repetition versus imagery were tested in separate blocks (which made performance for each strategy easier to monitor) compared to when items learned with each strategy were tested in an intermixed manner.

Two studies have specifically investigated the conditions under which people can learn from experience about the testing effect. Tullis et al. (2013) had learners study a set of word pairs and then practice the pairs using restudying or testing, make strategy predictions (i.e., estimate how many restudied versus tested pairs they would remember the next day), take a critical test on the pairs (after a 1-day delay), and then repeat the procedure with novel pairs. Most participants demonstrated a testing effect in their critical test performance, but their predictions—both before and after the critical test experience—were approximately equivalent for tested and restudied pairs, i.e., study–test experience alone was insufficient for learners to update their beliefs about the relative effectiveness of testing versus restudying. In a follow-up experiment, learners had more accurate strategy beliefs (i.e., appreciated the testing effect in their strategy predictions) when they received feedback delineating exactly how many pairs they had recalled through testing and through restudying on the critical test. More recently, Rivers et al. (2022) used a similar multicycle procedure, except some participants received correct-answer feedback during practice testing. This testing-with-feedback strategy resulted in an even larger testing effect on memory performance, and learners who recognized this differential performance updated their strategy beliefs.

Although simply experiencing a testing effect in critical test performance is insufficient, receiving feedback broken down by strategy and/or experiencing a larger testing effect can promote accurate beliefs about testing versus restudying. However, even under these supportive conditions, learners consistently underestimated the beneficial effect of practice testing on their memory performance. The fact that study–test experience was not fully effective at correcting learners’ beliefs about practice testing emphasizes the importance of investigating other approaches (or combinations of approaches) to improve learners’ strategy beliefs.

### 1.4. Combining Test Experience and Direct Instruction

Would the combination of experiencing a testing effect on one’s own memory *and* being instructed about the benefits of practice testing lead to more accurate strategy beliefs? Although no research to date has directly compared these two debiasing techniques for the testing effect, interventions that involve both (1) instructing learners about the effectiveness of particular strategies, and (2) experiencing the consequences of using (vs. not using) practice testing on memory performance have been found to lead to greater endorsement and use of the strategy (e.g., Biwer et al. 2020; Einstein et al. 2012; Hotta et al. 2014; Hui et al. 2021a, 2022; for a theoretical framework, see McDaniel and Einstein 2020). Though the results from these investigations are encouraging, they cannot answer the key question of the current research—that is, they do not offer a direct comparison of each debiasing technique, so it remains an open question whether both techniques combined will lead to greater benefits than either technique alone. Based on prior research, I predicted that both techniques would be effective in changing learners’ beliefs about practice testing. In addition, given that both experience and instruction may play different roles in correcting beliefs, I predicted that the combination of both techniques would be more effective than either technique alone. 

Participants were randomly assigned to one of three groups. Participants in the test experience group underwent a multicycle study–test procedure similar to that used in prior research that was shown to promote accurate beliefs about practice testing (Rivers et al. 2022). In particular, participants studied unrelated word pairs, practiced half the pairs through restudying and half through testing (with correct-answer feedback), completed a critical test on the pairs after a 1-day delay, and then received feedback regarding their test performance by strategy type. Participants in the direct instruction group read a short passage on the purported benefits of practice testing over restudying for memory; this passage explained a sample testing effect procedure (with word pairs), described the results and a brief explanation, acknowledged that restudying feels easier than practice testing during acquisition, and addressed learners’ possible misconception that testing is solely a monitoring tool. Finally, participants in the experience + instruction group experienced both debiasing techniques. All participants made global-differentiated predictions about memory for tested versus restudied pairs both before and after debiasing, and such predictions were then compared for each of the three groups.

## 2. Materials and Methods

### 2.1. Participants

The target sample size was 210 participants (70 participants per group). This sample size was based on an a priori power analysis conducted using G*Power (Faul et al. 2007). For this analysis, power was set at 0.80 and one-tailed α = 0.05 to detect a small difference (Cohen’s *d* = 0.30) between predictions for tested versus restudied material.

Participants were recruited via Prolific Academic, a platform for crowdsourcing participants for behavioral research known to produce higher-quality data (e.g., participants failing fewer attention checks) compared to other platforms such as Amazon’s Mechanical Turk (Palan and Schitter 2018; Peer et al. 2017). Prior survey research has found that online participants hold similar beliefs about learning strategies as compared to college students (Yan et al. 2014). 

Participants (*M*_age_ = 25.38 years, 60% male, 63% current students) were fluent in English; had an approval rate of 90% or higher on prior Prolific studies; and received compensation of USD $5.50 if they completed the entire experiment (which took approximately 20 min for session 1 and 30 min for session 2) and submitted a valid completion code. Data were analyzed from 209 participants. An additional 125 participants were dropped from the analysis because of exclusion criteria, the details of which are described below.

### 2.2. Design

Participants were randomly assigned to three groups: test experience (*n* = 70), direct instruction (*n* = 68), or experience + instruction (*n* = 71). Practice activity (testing vs. restudying) and learning cycle (1 vs. 2) were manipulated within participant.

### 2.3. Materials

Learning materials were 64 unrelated English word pairs composed of two nouns (e.g., trout–raccoon) from Tullis et al. (2013). The lack of association between cues and targets was confirmed by referring to prior norms (Nelson et al. 2004). These pairs were randomly split into two counterbalanced lists. One list was presented during Cycle 1 and the other was presented during Cycle 2, with the order of presentation counterbalanced across participants. Within these lists, half of the pairs were randomly assigned to the testing condition, and half were assigned to the restudy condition, with the pairs counterbalanced across participants such that they were equally likely to appear in either practice activity.

### 2.4. Procedure

**Figure 1:**
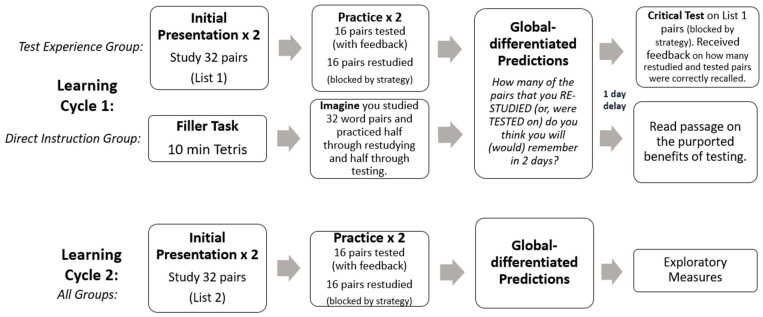
Overview of procedure used in the current investigation. Participants in the experience + instruction group gained study/practice/test experience during Cycle 1. Following the test on List 1, they also read the paragraph about the benefits of practice testing.

The experiment was completed online via Qualtrics Survey Software. An overview of the procedure is displayed in Figure 1. All participants underwent two learning cycles. The procedure for the first learning cycle depended on group assignment, whereas the second learning cycle was the same for all participants. Further details are provided below.

#### 2.4.1. Cycle 1 by Group Assignment

##### Test Experience Group

During the initial presentation phase, participants were presented with 32 word pairs (List 1). Each pair was presented for 4 s. Participants underwent this presentation phase twice. 

Following the presentation, participants engaged in a practice phase during which they restudied half of the pairs and were tested on the other half in two separate blocks (to make the two practice activities more salient; Yan et al. 2016). In the restudy condition, each pair was again presented for 4 s. In the testing-with-feedback condition, participants attempted to recall the target word (e.g., raccoon) when presented with the cue word (e.g., trout—?). Participants were given unlimited time to respond. After attempting recall, the cue and target were presented for 2 s.

After practicing all 32 pairs, participants made global-differentiated predictions for tested and restudied pairs (i.e., “From the 16 word pairs that you RESTUDIED [or, were TESTED on], how many do you think you will remember tomorrow?”). Participants were given unlimited time to respond with a value between 0–16, and the question order was counterbalanced across participants.

Approximately 24 h later, participants completed a critical cued-recall test on all List 1 pairs. Participants were tested on all the pairs they previously restudied and all the pairs they were previously tested on in two separate blocks (as in Price et al. 2008). The testing order was counterbalanced across participants such that half of the participants encountered previously restudied pairs first, whereas half of the participants encountered previously tested pairs first. No feedback was provided during the critical test.

Following the critical test of both previously tested and restudied pairs, participants were told the total number of restudied and tested pairs they had correctly recalled. This feedback remained visible until participants chose to advance (though they were required to remain on the screen for at least 10 s). To ensure participants processed the feedback, they were then asked (on the next screen) to choose which statement was true for them: That they recalled more tested than restudied pairs, that they recalled more restudied than tested pairs, or that they recalled an equal number of tested and restudied pairs.

Participants then completed an attribution questionnaire modified from Yan et al. (2016; see also Pan and Rivers 2023). Participants responded to the question, “What do you believe is the most important reason for your better performance on the tested or restudied pairs?” They were asked to select from four options: “I made lucky guesses”, “That strategy was more effective for learning than the other strategy”, “That particular set of pairs was easier than the other pairs”, or “Other reason”.

##### Direct Instruction Group

In contrast to the participants in the test experience group, who actually gained experience with the study, practice, and critical test procedure, participants in the direct instruction group completed a brief filler task (10 min of the game Tetris) to equate for study/practice time. They were then asked to imagine they had studied a set of 32 word pairs twice, then practiced half (i.e., 16) through testing with feedback and half through restudying; examples were provided. Then, they made global-differentiated predictions as if they were going to take a test one day later.

One day later, they read a ~500-word passage on the purported benefits of practice testing (modified from Ariel and Karpicke 2018; Bernacki et al. 2020; Yan et al. 2016); see Appendix A. Before reading the passage, they were told to read carefully because they may be tested on the content later. Participants had unlimited time to read the passage but were required to stay on the page for at least 30 s.

Immediately after reading the passage, participants received a short multiple-choice test on the content on the subsequent screen (Appendix B). If they did not provide correct responses, they were told to reread the passage and again received the same test; this procedure only occurred one time regardless of performance on the second test.

##### Experience + Instruction Group

Like participants in the test experience group, participants in the experience + instruction group gained study/practice/critical test experience during Cycle 1. Following the critical test (and feedback), they also read (and were tested on) the passage about the benefits of testing as in the direct instruction group.

#### 2.4.2. Cycle 2 and Exploratory Measures

Following the Cycle 1 procedure described above, all participants underwent a second learning cycle that involved studying a list of 32 word pairs (different from those learned in Cycle 1, if applicable), practicing half of the pairs through testing and half through restudying, and making global-differentiated predictions as if they were going to take a test in two days. The procedure for this cycle was identical to that of Cycle 1 of the test experience group.

Following predictions, participants completed a few exploratory measures. First, they were asked to rate the effectiveness of restudying and testing with feedback for learning word pairs on a scale from 1 (not effective) to 10 (very effective). The second measure was intended to assess whether learners’ beliefs about practice testing and restudying extended beyond learning word pairs. Participants read a hypothetical learning scenario that described two classes that were presented with a prose passage (modified from McCabe 2011). In one class (Class A), the students studied the passage twice and in the other (Class B), the students studied the passage and then were asked to write down as much as they could remember from the passage. Participants rated on a 7-point scale the extent to which they predicted the two classes would perform on a final test on the passage 7 days later. A rating of “1” indicated that Class A would perform better, a neutral rating of “4” would indicate that both classes would have equivalent test scores, and a rating of “7” would indicate that Class B would perform better. Finally, participants rated on a 7-point scale the extent to which they were likely to use rereading/restudying and testing with feedback when preparing for course exams (hypothetically, if the participant was not currently a student) compared to before they had participated in the experiment. A rating of “1” indicated that they were less likely to use the strategy compared to before participating in the experiment, a neutral rating of “4” would indicate that they were equally likely, and a rating of “7” would indicate that they were more likely.

At the end of the experiment, participants were asked if they had ever learned about the benefits of practice testing (compared to restudying) for memory before participating in the experiment. Thirty-eight percent of participants responded “yes”, 44% responded “no”, and 18% responded “I’m not sure”. These percentages were roughly equivalent for each group. For participants who responded “yes” to this question, their global-differentiated predictions on Cycle 1 did not significantly differ for tested versus restudied pairs (*t* [79] = 1.29, *p* = 0.20).

## 3. Exclusion Criteria

Several exclusion criteria were applied to ensure quality data. First, data were not analyzed for participants who failed to complete session 2 within 23 to 50 h after completing session 1 (*n* = 37). Second, several attention checks (no more than 8 per session) were included throughout the experiment. During blocks that involved studying (or restudying) word pairs, participants were occasionally asked to respond to a multiple-choice question (e.g., choose the word “lion” below) within 4 s. During blocks that involved testing (practice testing or the Cycle 1 critical test), participants were occasionally asked to type a specific word (e.g., “august”). Participants who did not correctly respond to the majority (i.e., at least 62%) of the attention checks in each session were excluded from the analysis (*n* = 18). Third, participants in the test experience groups who did not recall at least two pairs on the final criterion test (6% of the total pairs) were excluded from the analysis (*n* = 12). Fourth, participants in the direct instruction groups who did not provide at least three out of the four responses about the passage about testing (after the second reading) were excluded from the analysis (*n* = 17). Most participants (81%) correctly responded to all four responses. Fifth, participants in the test experience groups were told the total number of restudied and tested pairs they had correctly recalled after the critical test and then asked to choose the correct statement about their recall performance (given the following options: “I recalled more tested than restudied pairs”, “I recalled more restudied than tested pairs”, or “I recalled an equal number of tested and restudied pairs”). Participants who responded incorrectly to this question were excluded from the analysis (*n* = 28), given they did not accurately process the feedback provided. Finally, at the end of each session, participants were asked if they had any technical difficulties during the experiment or if they had cheated, and were excluded from analysis if they had significant technical difficulties (e.g., internet outage during study; *n* = 3) or reported cheating of any kind (e.g., taking notes on the pairs presented; *n* = 10).

In summary, 46 participants were excluded from the test experience group, 25 participants were excluded from the direct instruction group, and 54 participants were excluded from the experience + instruction group.

## 4. Results

Effect sizes for *t*-tests are reported in terms of Hedges’ *g* (formulas from Lakens 2013).

### 4.1. Memory Performance

Responses on tests were marked as correct if they matched the target exactly. During Cycle 1, only participants in the test experience and experience + instruction groups (*n* = 141) engaged in practice. Across both groups, the mean number of pairs recalled (out of 16) during the practice test phase during Cycle 1 was 7.35 (*SE* = 0.40) for Round 1 and 9.87 (*SE* = 0.38) for Round 2. During Cycle 2, all participants engaged in practice. Across these three groups, the mean number of pairs recalled during the practice test phase during Cycle 2 was 8.16 (*SE* = 0.32) for Round 1 and 10.62 (*SE* = 0.31) for Round 2.

**Figure 2:**
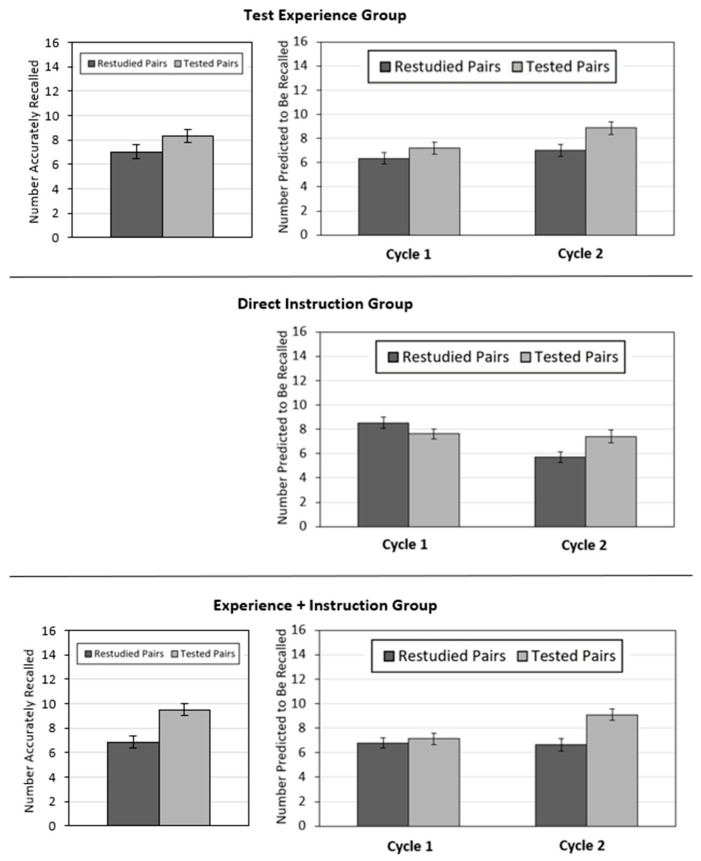
Number of pairs recalled on the critical test as a function of practice activity (**left panels**) and number of pairs predicted to be recalled during learning Cycles 1 and 2 as a function of practice activity (**right panels**) for each group.

Only participants in the test experience and experience + instruction groups underwent the critical test. The 2 (group: test experience, experience + instruction) × 2 (practice activity: restudying, testing with feedback) ANOVA on critical test performance revealed a significant interaction, *F*(1, 139) = 4.80, *p* = 0.03, η_p_^2^ = 0.03. Participants in both groups demonstrated a testing effect, but this effect was larger for the experience + instruction group (presented on the left side of Figure 2), i.e., participants in the test experience group recalled a greater number of tested than restudied pairs, *t*(69) = 2.72, *p* = 0.008, 95% CI [0.34, 2.23], *g*_av_ = 0.40. Of these 70 participants, 43 demonstrated a testing effect, 11 demonstrated no differences between restudied and tested pairs, and 16 demonstrated an advantage for restudied over tested pairs. Participants in the experience + instruction group also recalled a greater number of tested than restudied pairs; *t*(70) = 6.53, *p* < 0.001, 95% CI [1.84, 3.46], *g*_av_ = 0.61. Of these 71 participants, 48 demonstrated a testing effect, 9 demonstrated no differences between restudied and tested pairs, and 14 demonstrated an advantage for restudied over tested pairs.

### 4.2. Global-Differentiated Predictions

Participants’ global-differentiated predictions are presented on the right side of Figure 2. Patterns of predictions for tested and restudied pairs differed in Cycle 1 based on group assignment. Thus, I conducted a 3 (group) × 2 (cycle) × 2 (practice activity) ANOVA on global-differentiated predictions. This analysis revealed a significant 3-way interaction, *F*(2, 206) = 3.57, *p* = 0.03, η_p_^2^ = 0.03. To follow-up this significant interaction, I present the 2 (cycle) × 2 (practice activity) ANOVAs for each group below. To foreshadow, the 2 × 2 interaction was significant for all three groups.

#### 4.2.1. Test Experience Group

A 2 (learning cycle) × 2 (practice activity) repeated-measures ANOVA revealed a main effect of learning cycle, *F*(1, 69) = 8.92, *p* = 0.004, η_p_^2^ = 0.11, and of practice activity, *F*(1, 69) = 25.49, *p* < 0.001, η_p_^2^ = 0.27, which were qualified by a significant interaction, *F*(1, 69) = 6.84, *p* = 0.011, η_p_^2^ = 0.09. For both cycles, participants made higher predictions for tested than restudied pairs, but this difference was larger for Cycle 2, *t*(69) = 5.10, *p* < 0.001, 95% CI [1.11, 2.54], *g*_av_ = 0.43, than for Cycle 1, *t*(69) = 2.87, *p* = 0.005, 95% CI [0.25, 1.40], *g*_av_ = 0.20.

The same pattern of results was found when this analysis was restricted to only include participants who showed a testing effect in their recall performance (i.e., the interaction was still significant, *F*(1, 42) = 13.98, *p* = 0.001, η_p_^2^ = 0.25). However, when the analysis was restricted to only include participants who did *not* show a testing effect, the interaction was no longer significant, *F*(1, 26) = 0.55, *p* = 0.47, η_p_^2^ = 0.02. Participants’ global-differentiated predictions conditionalized by their performance on the critical test are presented in Table 1.

#### 4.2.2. Direct Instruction Group

The 2 (learning cycle) × 2 (practice activity) ANOVA revealed a main effect of learning cycle, *F*(1, 67) = 12.69, *p* = 0.001, η_p_^2^ = 0.16, but the main effect of practice activity was not significant, *F*(1, 67) = 2.88, *p* = 0.094, η_p_^2^ = 0.04. The 2 × 2 interaction was significant, *F*(1, 67) = 29.11, *p* < 0.001, η_p_^2^ = 0.30. During Cycle 1, participants made higher predictions for pairs that were restudied versus tested, *t*(67) = 2.55, *p* = 0.005, 95% CI [0.20, 1.62], *g*_av_ = 0.20, but during Cycle 2, participants made higher predictions for pairs that were tested versus restudied, *t*(67) = 5.51, *p* < 0.001, 95% CI [1.08, 2.30], *g*_av_ = 0.42.

#### 4.2.3. Experience + Instruction Group

The 2 (learning cycle) × 2 (practice activity) ANOVA revealed a main effect of learning cycle, *F*(1, 70) = 10.02, *p* = 0.002, η_p_^2^ = 0.13, and practice activity, *F*(1, 70) = 35.16, *p* < 0.001, η_p_^2^ = 0.33, which were qualified by a significant interaction, *F*(1, 70) = 23.73, *p* < 0.001, η_p_^2^ = 0.25. During Cycle 1, predictions did not differ for pairs that were tested versus restudied, *t*(70) = 0.94, *p* = 0.35, 95% CI [−0.38, 1.06], *g*_av_ = 0.09, but during Cycle 2, participants made higher predictions for pairs that were tested versus restudied, *t*(70) = 9.04, *p* < 0.001, 95% CI [1.90, 2.97], *g*_av_ = 0.61.

The same pattern of results was found when this analysis was restricted to only include participants who showed a testing effect in their recall performance—that is, the interaction was still significant, *F*(1, 47) = 15.10, *p* < 0.001, η_p_^2^ = 0.24. In addition, when the analysis was restricted to only include participants who did *not* show a testing effect, the interaction was still significant, *F*(1, 22) = 8.65, *p* = 0.008, η_p_^2^ = 0.28. Participants’ global-differentiated predictions conditionalized by their performance on the critical test are presented in Table 1.

### 4.3. Exploratory Measures

#### 4.3.1. Ratings of Strategy Effectiveness

Participants were asked to rate the effectiveness of restudying and testing with feedback for learning word pairs on a scale from 1 (not effective) to 10 (very effective). A 2 (practice activity) × 3 (group) mixed ANOVA was conducted on these ratings. The main effect of practice activity was significant, *F*(1, 206) = 230.28, *p* < 0.001, η_p_^2^ = 0.53, with participants making higher ratings for testing with feedback (*M* = 7.91, *SE* = 0.11) than restudying (*M* = 5.56, *SE* = 0.13). The interaction was not significant, *F*(2, 206) = 1.33, *p* = 0.27, η_p_^2^ = 0.01, suggesting that on average, participants in all three groups rated testing with feedback as more effective than restudying for learning word pairs at the end of the experiment.

#### 4.3.2. Hypothetical Learning Scenario

As a reminder, participants read a hypothetical learning scenario that described two classes of students that were presented with a prose passage, then either reread the passage or took a practice test on the passage, then took a delayed final test on the passage (see McCabe 2011). On the 7-point scale, with values greater than 5 indicating better long-term memory performance for those who took a practice test, participants provided an average response of 5.71 (*SE* = 0.08), and this value did not differ by group assignment, *F*(2, 206) = 1.74, *p* = 0.18. Overall, 90.4% of participants provided the “correct” response of 5 or higher. Thus, the vast majority of participants perceived a learning advantage for testing over restudying, even for prose material.

#### 4.3.3. Ratings of Likelihood of Use

On a 7-point scale, participants rated the extent to which they were likely to use restudying and testing with feedback when preparing for exams compared to before they had participated in the experiment. For restudying, participants provided an average response of 4.07 (*SE* = 0.11), but this value differed by group assignment; *F*(2, 206) = 4.85, *p* = 0.009. On average, those in the test experience group rated that they were more likely to use restudying compared to before they had participated in the experiment (*M* = 4.51, *SE* = 0.20), whereas those in the experience + instruction group rated that they were less likely to use restudying compared to before participating in the experiment (*M* = 3.66, *SE* = 0.20); *t*(139) = 3.01, 95% CI [0.28, 0.85], *g*_s_ = 0.50. No other contrasts were significant, including comparisons with the direct instruction group, who were rated that they were equally likely to use restudying compared to before they participated in the experiment (*M* = 4.03, *SE* = 0.18). Overall, 44% of participants rated that they were less likely to use restudying compared to before participating in the experiment, 14% were equally likely, and 42% were more likely.

For testing with feedback, participants provided an average response of 5.55 (*SE* = 0.09), and this value did not differ by group; *F*(2, 206) = 3.97, *p* = 0.11. Overall, 9% of participants rated that they were less likely to use testing with feedback compared to before participating in the experiment, 6% were equally likely, and 85% were more likely.

Taken together, participating in the experiment positively influenced participants’ likelihood to use testing with feedback when preparing for exams, whereas the experiment did not have as much of an impact on participants’ likelihood to use restudying.

#### 4.3.4. Attributions of Critical Test Performance

**Figure 3:**
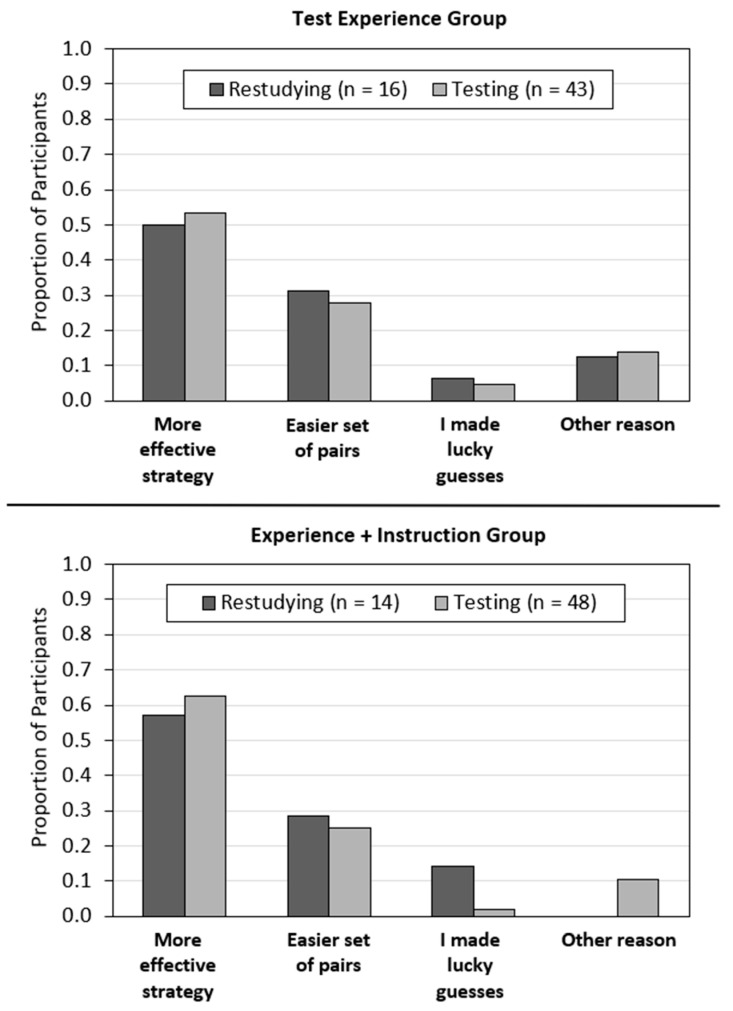
Attribution outcomes for the test experience group (**top panel**) and the experience + instruction group (**bottom panel**). Participants’ attributions for why one strategy was better for learning than the other in relation to whether restudying was better or testing was better in their critical test performance.

Participants’ attributions for their performance on the critical test are presented in Figure 3. As a reminder, participants in the test experience and experience + instruction groups were asked to provide the most important reason why they performed better on pairs that were tested or restudied on the critical test. Participants who performed equally well on tested and restudied pairs (*n* = 20) were not included in this analysis.

Most participants who experienced a testing effect in their critical test performance (*n* = 91) attributed their performance to a differential effectiveness of the practice strategy (58%); the remainder of the responses reflected a (mistaken) belief that the pairs in one condition were easier than the other (26%), lucky guesses (3%), and other reasons (12%). Participants in these groups who experienced a recall advantage for restudied pairs on the critical test (*n* = 30) attributed their performance to a differential effectiveness of the practice strategy (53%), a belief that the pairs in one condition were easier than the other (30%), lucky guesses (10%), and other reasons (7%). These patterns of attributions did not differ for the test experience versus test + instruction groups.

## 5. Discussion

The current experiment investigated the extent to which learners’ beliefs about the testing effect could be improved via test experience, direct instruction, and a combination of both techniques. Although prior research has investigated the effectiveness of these debiasing techniques for the testing effect and other learning strategies (e.g., McCabe 2011; Rivers et al. 2022; Tullis et al. 2013; Yan et al. 2016), no investigation has directly compared their effectiveness. Based on prior research, I expected participants who experienced a testing effect on their own memory performance (test experience group) and participants who read a passage about the purported benefits of practice testing (direct instruction group) would update their strategy beliefs (and thus their metacognitive judgments) across learning cycles. In addition, given these two debiasing techniques may play different roles in correcting learners’ beliefs about the relative effectiveness of practice testing and restudying, I expected the experience + instruction group to be more effective than either technique alone. Across all three groups, a significant interaction was found between practice activity and learning cycle. Participants in all three groups updated their beliefs about the relative effectiveness of practice testing and restudying across cycles (Figure 2). Comparing the effect sizes across the groups, a greater interaction effect was found for the direct instruction group (η_p_^2^ = 0.30) and experience + instruction group (η_p_^2^ = 0.25) compared to the test experience group (η_p_^2^ = 0.09). However, a few caveats make it difficult to make definitive claims about which debiasing technique is most effective for correcting learners’ beliefs about testing.

First, the current investigation did not include a control group in which learners did not undergo any debiasing intervention, but simply made predictions about memory performance for restudying and practice testing at two different time points. It is possible that over time, learners would have adjusted their beliefs about strategy effectiveness even without test experience or instruction. This possibility is unlikely given prior research suggesting that learners hold inaccurate beliefs about the testing effect even after study–test experience (without feedback or other forms of scaffolding; Tullis et al. 2013). Nevertheless, the design of the current investigation precludes definitive causal claims about the impact of the various debiasing techniques on learners’ beliefs.

Second, on the critical test, a larger testing effect in recall was found for the experience + instruction group (*g*_av_ = 0.61) compared to the test experience group (*g*_av_ = 0.40). The procedure for these groups was the same until the critical test, so this variation in the testing effect was likely due to noise or differential attrition between the two groups. However, because a larger effect on memory can contribute to a greater degree of knowledge updating across learning cycles (Mueller et al. 2015; Rivers et al. 2022), what is driving the difference in strategy beliefs for these two groups is unclear.

Third, participants in the three groups started with different patterns of Cycle 1 predictions about the relative effectiveness of the two learning strategies. In particular, during Cycle 1, the direct instruction group made higher predictions for restudying than testing, the test experience group made higher predictions for testing than restudying, and the experience + instruction group made equivalent predictions for the two practice activities. This variation in learners’ initial beliefs about the relative effectiveness of the two strategies is unsurprising. In prior investigations exploring learners’ beliefs about testing, some have found that participants believe restudying is more effective than testing, whereas others have found that participants believe the two strategies are equally effective (Rivers 2021). Thus, the difference in beliefs could simply be due to noise. Another possibility is that participants in all groups begin the experiment believing that restudying is more effective than testing, but the study/practice experience during Cycle 1—a procedure only experienced by the test experience groups—influences learners’ beliefs about the relative effectiveness of the two learning strategies (such that they believe the two strategies are equally effective, or even that testing is more effective than restudying).

Practically speaking, any intervention aimed at addressing learners’ beliefs about practice testing should result in (more) accurate beliefs about the strategy, i.e., regardless of learners’ beliefs before administering an intervention, learners should believe that testing is more effective than restudying for long-term memory after the intervention. It appears that all three debiasing techniques were effective in this regard. Specifically, a 2 (practice activity: restudying, testing with feedback) × 3 (group: test experience, direct instruction, experience + instruction) mixed ANOVA was conducted on Cycle 2 predictions. Cycle 2 predictions were higher for tested pairs (*M* = 8.45, *SE* = 0.29) than restudied pairs (*M* = 6.47, *SE* = 0.27) for all three groups, as indicated by the main effect of practice activity, *F*(1, 206) = 120.34, *p* < 0.001, η_p_^2^ = 0.37. However, the interaction was not significant, *F*(2, 206) = 1.61, *p* = 0.20, η_p_^2^ = 0.02, i.e., participants’ beliefs about the differential effectiveness of the two strategies were similar following all three debiasing techniques.

Results from the exploratory measures were also consistent with the Cycle 2 prediction outcomes. In particular, when participants were directly asked to rate the effectiveness of testing and restudying for learning word pairs, they consistently rated testing with feedback as more effective than restudying. Whereas metacognitive judgments are influenced by variables other than perceived strategy effectiveness (e.g., perceived difficulty of the words pairs, ease of retrieval, etc.), this questionnaire provided a more direct measure of strategy beliefs (for a similar argument, see Price et al. 2008), providing further confidence in the main conclusions. In addition, at the end of the experiment, participants indicated that testing would be more effective than restudying for learning a text passage and that they were more likely to use testing when preparing for an exam. Taken together, the results of this experiment suggest that all three debiasing techniques—test experience, direct instruction, and their combination—can lead to accurate beliefs about the testing effect.

### 5.1. Conditions That Promote Accurate Strategy Beliefs

The fact that the combination of both test experience and direct instruction was not more effective than either technique alone was somewhat surprising. Specifically, instruction may be well-suited to address learners’ a priori beliefs (and potential misconceptions) about testing (e.g., that testing is a tool for monitoring learning, but not for improving memory), whereas test experience may be better suited to convince learners that the practice strategy is effective for their own long-term memory. In addition, both experience and instruction have strengths and weaknesses as general techniques for correcting beliefs (for a general discussion, see Lee and Anderson 2013), and thus their combination may be the most compelling.

For example, test experience may only be effective at changing learners’ beliefs when certain conditions are met—i.e., when participants actually experience a testing effect in their performance and attribute this effect to the strategies themselves (e.g., Dunlosky and Hertzog 2000). Indeed, participants in the test experience group only updated their beliefs (i.e., made higher predictions for tested than restudied pairs during Cycle 2) when they experienced a testing effect on the critical test. In addition, of the participants who demonstrated a testing effect (in either the test experience or experience + instruction group), the differential effect for Cycle 2 predictions (e.g., prediction for tested pairs − prediction for restudied pairs) was larger for those who attributed their performance to the testing strategy (*M*_diff_ = 3.66) compared to those who attributed their performance to lucky guesses, an easier set of pairs, or something else (*M*_diff_ = 1.74).

Direct instruction may only be effective under certain conditions, too. In particular, for the instruction to be effective at changing learners’ relative beliefs about strategies, it needs to be believed (e.g., Rich et al. 2017). Given that the direct instruction group updated their beliefs across cycles, it appears that learners did believe the passage they read about testing. However, the demand characteristics were likely heavier for this group than the test experience group—it seems reasonable that after reading the passage about practice testing, participants in the direct instruction group would infer that the goal of the experiment was for them to use the information from the passage to inform their predictions and ratings of strategy effectiveness. The instruction was also presented close in time to the measure of beliefs in Cycle 2, so participants’ predictions could have been influenced by the recency of the instructional intervention. Thus, future research should investigate the effectiveness of direct instruction about practice testing in other contexts and across longer periods of time to ensure that the updated beliefs following instruction are not merely a result of participant acquiescence, recency, or other factors unique to the methods used in this investigation.

As long as the instruction provided is believed by participants, the combination of instruction and test experience may be effective at updating learners’ beliefs regardless of their recall or performance attributions. In particular, for those who experience a restudy benefit or no recall difference between tested and restudied pairs, reading about the benefits of testing might lead learners to update their strategy beliefs when they may not have otherwise. Comparing the results for the test experience and experience + instruction groups, this idea was supported (Table 1). Specifically, conditional analyses revealed that participants who did not demonstrate a testing effect in their critical test performance did not update their strategy beliefs in the test experience group, but they did in the experience + instruction group (i.e., the cycle × practice activity interaction was still significant when the analysis was restricted to only include participants who did not show a testing effect in their recall).

Overall, why wasn’t the combination of experience + instruction more effective than the two techniques on their own? One possibility is that learners already hit a functional ceiling in the test experience and direct instruction groups, and thus the combination of the two techniques would not provide any additional advantages to learners’ beliefs. Some evidence supports this idea: the extent to which learners judged testing as more effective than restudying in their Cycle 2 predictions was similar to recall performance on the critical test. On average, participants in the test experience group experienced a medium testing effect (i.e., *g*_av_ = 0.40). In addition, the size of the testing effect they expected for a novel set of word pairs (based on their Cycle 2 predictions) was comparable (i.e., *g*_av_ = 0.43). Because predictions were already aligned with performance, instruction may not have incurred any additional benefits. Assessing the alignment of beliefs with actual performance for the direct instruction group is not possible because they did not experience a critical test. Nevertheless, their predicted testing effect (*g*_av_ = 0.42) was similar to the test experience group’s testing effect. Finally, the experience + instruction group experienced a medium-large testing effect in their critical test performance (*g*_av_ = 0.61) and predicted a similar-sized effect in their Cycle 2 predictions (*g*_av_ = 0.61). Thus, at least on average, participants in all three groups made predictions that were in line with their recall (or likely recall) performance.

### 5.2. Educational Implications and Future Directions

The current investigation identified two techniques that lead to accurate beliefs about the value of practice testing for learning. Accordingly, experiencing the effectiveness of practice testing on one’s own memory performance and receiving instruction about its benefits may be critical components of interventions designed to improve students’ beliefs about and self-regulated use of practice testing (e.g., McDaniel and Einstein 2020). Future research should continue to investigate the conditions under which each of these debiasing techniques is effective at changing learners’ beliefs for practice testing and other learning strategies such as interleaving (e.g., Sun et al. 2022), particularly across longer time periods.

That said, simply believing a particular strategy is effective may be insufficient to promote lasting behavior change (e.g., Foerst et al. 2017). In particular, recent investigations have revealed additional barriers that students face when implementing effective learning strategies such as practice testing, including uncertainty about how to use the strategies and concerns about how much time and effort they require (Biwer et al. 2020; Rea et al. 2022). Accordingly, in addition to addressing strategy beliefs, interventions aimed at promoting students’ self-regulated use of effective learning strategies should also consider factors such as perceived effort (Dunlosky et al. 2020), motivation (Zepeda et al. 2020), time management (Wolters and Brady 2020), and contextual cues that contribute to students’ study habits (Fiorella 2020). Indeed, interventions that address multiple barriers to self-regulated learning show promise for student success (e.g., Biwer et al. 2020, 2023; McDaniel et al. 2021).

## 6. Conclusions

Identifying the conditions under which students can appreciate the benefits of practice testing—a highly robust learning strategy—is an important first step toward informing interventions aimed at improving self-regulated learning and achievement. The results of the present investigation suggest that, at least under certain conditions, test experience, direct instruction, and their combination can lead to accurate beliefs about the relative effectiveness of practice testing versus restudying for long-term retention.

## Figures and Tables

**Table 1:** Global-differentiated predictions by performance.

	Restudied Pairs *M* (*SE*)	Tested Pairs *M* (*SE*)	*t*	*p*
Test Experience Group				
Testing Effect on Performance (*n* = 43)				
Cycle 1	6.21 (0.57)	7.30 (0.60)	2.77	.008
Cycle 2	5.81 (0.52)	8.77 (0.65)	6.41	<.001
No Testing Effect on Performance (*n* = 27)				
Cycle 1	6.59 (0.82)	7.00 (0.86)	1.02	.32
Cycle 2	8.96 (0.83)	9.00 (0.86)	0.10	.92
Experience + Instruction Group				
Testing Effect on Performance (*n* = 48)				
Cycle 1	7.06 (0.54)	7.69 (0.58)	1.47	.15
Cycle 2	6.96 (0.61)	9.73 (0.52)	7.49	<.001
No Testing Effect on Performance (*n* = 23)				
Cycle 1	6.17 (0.50)	5.91 (0.86)	0.39	.70
Cycle 2	6.00 (0.75)	7.74 (0.86)	6.48	<.001

Note. Testing Effect in Performance = Participants who performed better on tested than restudied pairs on the critical test. No Testing Effect in Performance = Participants who either recalled an equivalent number of tested and restudied pairs or recalled more restudied than tested pairs on the critical test. *SE* = Standard error of each mean. *t* = Two-tailed paired-samples *t*-test comparing predictions for tested and restudied pairs.

## Data Availability

The data presented in this investigation are openly available in the Open Science Framework at https://osf.io/tq6sh/ (accessed on 20 July 2023). DOI: 10.17605/OSF.IO/TQ6SH.

## Appendix A. Instruction about the Benefits of Practice Testing for Memory

Note. Data presented in the figure were from the large testing effect group of Experiment 3 of Rivers et al. (2022).

We want to tell you about a strategy that is extremely effective for improving your long-term memory for what you are studying: self-testing. Research shows that people remember more information when they test themselves than when they just reread or restudy, which is called the testing effect. The benefits of self-testing for memory are even larger when people receive feedback after testing.

To illustrate this effect, consider students who are presented with 32 word pairs to learn (e.g., dog–spoon). Their goal is to recall as many of the pairs as possible on a future “cued-recall” test in which they will be presented with the words on the left (e.g., dog–?) and asked to recall the words on the right side of the pairs (e.g., spoon).

The students first study each word pair two times, so that they can gain some initial familiarity with all of them. Next, the students practice half of the pairs through restudying and half of the pairs through testing, i.e., for 16 of the pairs, students restudy the pairs—they are re-presented with each pair again and have a chance to reread it to learn it. For the other 16 pairs, students practice retrieving the pairs through cued-recall (e.g., dog–?). On these tests, after the students attempt to recall an answer, they receive feedback on the correct answer (i.e., they are shown the response, spoon).

Two days later, the students receive a final cued-recall test on all the pairs they initially learned and practiced. The figure below shows memory performance on the final test for the pairs that were restudied versus tested. On average, students recalled 27% more of the material that was tested compared to the material that was restudied. Beyond this single demonstration, there have been hundreds of studies exploring the testing effect, using people of different ages and backgrounds, different kinds of materials (from simple pairs to complex text materials), and in real classrooms. In all these contexts, self-testing boosts people’s learning as compared to simply rereading or restudying the to-be-learned materials.

In educational settings, tests are often used to assess knowledge and to assign grades. Students typically use tests to assess what they know versus do not know. However, as described above, tests also serve an additional purpose: to help you remember the material you are tested on. Most people think that restudying is a better choice, but previous research has shown that an overwhelming 90% of people remember more after self-testing than restudying. This comes as a surprise to many people, as there is a strong sense that restudying feels easier during learning because the material is readily available. However, after restudying, the material is quickly forgotten. In contrast, testing helps make the material “stick” so that it is more likely to be remembered in the future.

## Appendix B. Test on Practice Testing Passage

Note. Correct answers are denoted with an asterisk (*).

1. Based on the research previously described, which strategy is more effective for learning word pairs (for example, dog–spoon)?
  - Self-testing*
  - Restudying
2. Select all that apply. Tests can be used to:
  - Help assess knowledge*
  - Help remember information*
3. In general, which strategy tends to feel easier during learning?
  - Self-testing
  - Restudying*
